# Multicenter prospective surveillance study of viral agents causing meningoencephalitis

**DOI:** 10.1038/s41598-021-86687-0

**Published:** 2021-03-30

**Authors:** Selda Hançerli Törün, Özge Kaba, Nurhayat Yakut, Eda Kepenekli Kadayıfçı, Manolya Kara, Mehpare Sarı Yanartaş, Ayper Somer, Burcu Bursal Duramaz, Özden Türel, Nazan Dalgıç, Emel Ekşi Alp, Enes Salı, Deniz Çakır, Pınar Önal, Haluk Çokuğraş, Fatma Deniz Aygün, Adem Karbuz, Mustafa Önel, Sevim Meşe, Ali Ağaçfidan

**Affiliations:** 1 Division of Pediatric Infectious Diseases, Department of Pediatrics, Istanbul Faculty of Medicine, Istanbul University, Istanbul, Turkey; 2 Division of Pediatric Infectious Diseases, Department of Pediatrics, Medical Faculty, Marmara University, Istanbul, Turkey; 3 Division of Pediatric Infectious Diseases, Department of Pediatrics, Bezmialem Vakif University Medical Faculty, Istanbul, Turkey; 4 Division of Pediatric Infectious Diseases, Şişli Etfal Training and Research Hospital, University of Health Sciences, Istanbul, Turkey; 5 Division of Pediatric Infectious Diseases, Ümraniye Training and Research Hospital, University of Health Sciences, Istanbul, Turkey; 6 Division of Pediatric Allergy, Immunology and Infectious Diseases, Department of Pediatrics, Cerrahpaşa Faculty of Medicine, Istanbul University-Cerrahpaşa, Istanbul, Turkey; 7 Division of Pediatric Infectious Diseases, Kanuni Sultan Süleyman Training and Research Hospital, University of Health Sciences, Istanbul, Turkey; 8 Division of Pediatric Infectious Diseases, Okmeydanı Training and Research Hospital, University of Health Sciences, Istanbul, Turkey; 9 Department of Microbiology, Faculty of Medicine, Istanbul University, Istanbul, Turkey

**Keywords:** Diseases, Health care

## Abstract

The frequency of bacterial factors causing central nervous system infections has decreased as a result of the development of our national immunization program. In this study, it is aimed to obtain the data of our local surveillance by defining the viral etiology in cases diagnosed with meningoencephalitis for 1 year. Previously healhty 186 children, who applied with findings suggesting viral meningoencephalitis to 8 different tertiary health centers between August 2018 and August 2019, in Istanbul, were included. The cerebrospinal fluid (CSF) sample was evaluated by polymerase chain reaction. The M:F ratio was 1.24 in the patient group, whose age ranged from 1 to 216 months (mean 40.2 ± 48.7). Viral factor was detected in 26.8%. Enterovirus was the most common agent (24%) and followed by Adenovirus (22%) and HHV type 6 (22%). In the rest of the samples revealed HHV type 7 (10%), EBV (6%), CMV (6%), HSV type 1 (6%), Parvovirus (4%) and VZV (2%). The most common symptoms were fever (79%) and convulsions (45.7%). Antibiotherapy and antiviral therapy was started 48.6% and 4% respectively. Mortality and sequela rate resulted 0.53% and 3.7%, respectively. This highlights the importance of monitoring trends in encephalitis in Turkey with aview to improving pathogen diagnosis for encephalitis and rapidly identifying novel emerging encephalitis-causing pathogens that demand public health action especially in national immunisation programme.

## Introduction

The concepts of encephalitis and meningitis, which are defined as brain parenchyma and inflammation of the meningeal membranes, are a pediatric emergency due to mortality and long-term sequelae potential^1^. These central nervous system (CNS) infections, which can cause confusing tables, have been collected under the definition of meningoencephalitis due to difficulties in making a clinical diagnosis^2^. The encephalitis picture accompanied by neurological dysfunction may also manifest with encephalopathy, fever, seizure, focal neurological deficits, and behavioral changes^3^.

Diversity in identifiable factors; It is thought to be caused by climate change, presence of epidemics, presence of atropod infections and changes in immunization programs^4^.

As a result of the development of our national immunization program, the frequency of bacterial agents causing central nervous system infections has been shown to decrease, and there are no data on viral factors. Turkey in the childhood immunization schedule in 13 of routine vaccination against the disease is carried out. These; Diphtheria, pertusis, tetanus, polio, hepatitis B, hepatitis A, H. influenzae type b, tuberculosis, measles, mumps, rubella, chickenpox and pneumococci. In this study, it is aimed to define the etiology in preliminary diagnosed cases of meningoencephalitis for a year and to discuss the possible diagnosis of the data of our local surveillance group with various parameters.

## Material and method

### Design

It was designed as a multicentric prospective observational study in which patients who will receive a diagnosis of viral meningoencephalitis will be evaluated by applying to eight different tertiary care institutions between August 2018 and August 2019 in Istanbul.

### Patients

One hundred and eighty-six patients, aged between 1 month and 18 years, who applied between August 2018 and August 2019 and were diagnosed with meningoencephalitis, participated in the study. The opinion of the International Encephalitis Consortium was based on in order to identify possible cases of meningoencephalitis. Any level of confusion, lethargy, and personality change lasting more than 24 h and requiring medical assistance were accepted as major criteria.Minor criteria were assessed as > 38° fever within 72 h or at the time of admission, generalized or partial seizures, new onset focal neurological findings, the presence of abnormal brain parenchyma in imaging, and the presence of abnormal activities in electroencephalography. In addition to the major criterion, 2 minor criteria were accepted for the definition of possible encephalitis, and ≥ 3 minor criteria were accepted for the definition of probable or confirmed encephalitis (3). The diagnosis was made by the pediatrician.

Patients with CNS shunt and patients with other CNS conditions such as intoxication, metabolic disease, stroke, vasculitis, trauma, autoimmune encephalitis were not included in the study. Patients under 1 month were not included, as there may be any disease that could mimic encephalitis. Being between a month and 18 years of age and obtaining informed consent form from both parents of the participant were among the criteria for inclusion in the study. The patient group that did not meet these criteria or whose lumbar puncture was contraindicated was excluded. After filling the forms which created for the study, the demographic, clinical, imaging and laboratory data obtained, and the data related to the treatment and treatment response were evaluated.

### Samples and laboratory analysis

Before antimicrobial treatment initiation, cerebrospinal fluid (CSF), complete blood count, C-reactive protein were obtained from patients who were diagnosed with meningoencephalitis and met the inclusion criteria. In order to determine viral etiology, 200 µl of CSF samples, which are allocated to ependorphs, were stored after by freezing at − 80 ℃. All of the samples were taken to the study simultaneously and by the same staff.

The CSF samples of the patients were studied with EZ1 Virus Mini Kit v2.0 (QIAGEN, Germany)in EZ1 Advanced XL (QIAGEN, Germany) extraction device in accordance with the package insert and nucleic acid isolates were obtained. These isolates were qualitatively studied in Multiplex Real-Time polymerase chain reaction (RT-PCR, FTD Neuro9, QIAGEN, Germany)devices (Rotor-Gene Q, QIAGEN, Germany) in accordance with the package insert.It is aimed to show the presence of DNA viruses as Cytomegalovirus (CMV), Ebstein Barr Virus (EBV), Herpes Simplex Virus (HSV) types 1 and 2, Varicella Zoster Virus (VZV), Human Herpes Virus (HHV) type 6 and 7, Adenovirus, Parvovirus B19 and RNA viruses as Parechovirus and Enterovirus.

### Statistical analysis

Categorical data are specified with numbers and percentages. Mean value and standard deviation were used in continuous data. Pearson chi-square test was used to compare the data of the group with and without an viraletiology. All statistical analyzes were done using SPSS version 23 program. Statistical value of p value < 0.05 was considered significant.

### Compliance with ethical statements

Ethics approval for the study was granted by Istanbul University Faculty of Medicine Clinical Research Ethics Committee (07/19/2018 ID 236404). All subjects gave written informed consent in accordance with the Declaration of Helsinki. Written informed consent was obtained from all participants, or from guardians or parents on behalf of participants under the age of 18 years.

## Results

**Figure 1:**
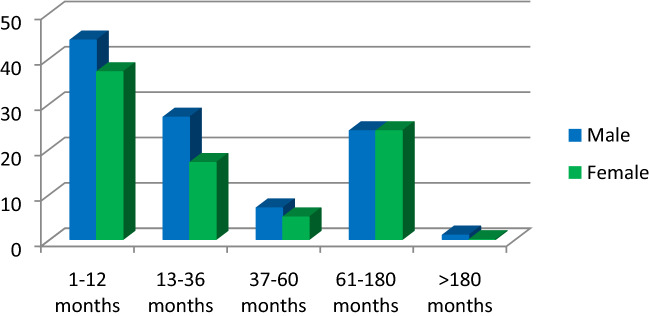
Distribution of participants by age group and gender.

The mean age of 186 patients included in the study was 40.2 ± 48.7 and the age range was 1–216 months (Fig. 1).

**Figure 2:**
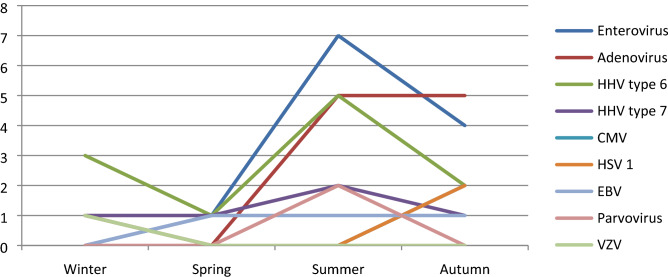
Application seasons of patients who are positive as a result of viral panel. *HHV* Human Herpes Virus, *CMV* Cytomegalovirus, *HSV* Herpes Simplex Virus, *EBV* Ebstein Barr Virus, *VZV* Varicella Zoster Virus.

In the patient group, the male/female ratio was 1.24. All of the patients were residing in the province of Istanbul and none of the patients had animal contact or travel history. When the applications made were examined seasonally, it was observed that applications were ratio with 30.1% (n = 56) in summer, 27.4% (n = 51) in autumn, 25.2% (n = 47) in winter and 17.2% (n = 32) in spring (Fig. 2).

It was seen that 82.3% (n = 153) of the patients were fully vaccinated according to their age with the national program. Data for the remaining patient group could not be obtained.

When the complaints of all patients were grouped, there was 79% (n = 147) fever, 45.7% (n = 85) convulsion, 12.4% (n = 23) diarrhea and 5.4% (n = 10) rash. At the time of admission, 28% (n = 52) of patients had complete loss of consciousness. The rates of detection of headache, ataxia, vomiting, cough, speech disorder, diplopia and abdominal pain, which are among other application findings, were 5.9%, 3.7%, 2.6%, 2.1%, 0.5%, 0.5% and 0.5%, respectively (Table 1).

**Table 1:** Distribution of age, gender and clinical presentations according to viral panel results.

	Enterovirus (n = 12)	Adenovirus (n = 11)	HHV type 6 (n = 11)	HHV type 7 (n = 5)		EBV (n = 3)	HSV type 1 (n = 3)	CMV (n = 3)	Negative (n = 136)
**Age**									
Mean ± SS	35.22 ± 41.47	36.54 ± 49.71	23.09 ± 37.36	73 ± 43.35	62 ± 64.81		15.66 ± 11.89	4.5 ± 2.12	42.16 ± 49.12
**Gender**									
M:F	1:1	3:8	9:2	2:3	2:1		1:2	1:2	19:15
**Clinicalpresentation**									
Fever	10	7	11	3	3		2	2	108
Convulsion	1	6	5	1	1		3	3	62
Diarrhea	0	2	2	0	0		0	0	19
Rash	0	0	2	1	0		0	0	6
Loss ofconsciousness	4	3	1	2	1		1	0	39
Ataxi	0	0	0	0	0		0	0	7
Headhache	3	0	1	2	0		0	0	6
Vomiting	2	0	1	0	0		0	0	2
Cough	0	0	0	0	0		0	0	4

*HHV* Human Herpes Virus, *CMV* Cytomegalovirus, *HSV* Herpes Simplex Virus, *EBV* Ebstein Barr Virus.

At least one viral factor was detected in 26.8% (n = 50) of the studied samples. Enterovirus ranked first with 24% (n = 12), followed by Adenovirus with 22% (n = 11) and 22% (n = 11) HHV type 6 (n = 11). The distribution of the detected factors according to the seasons can be seen in Fig. 2. In the rest of the examples, HHV type 7 (10%, n = 5), EBV (6%, n = 3), CMV (6%, n = 3), HSV type 1 (6%, n = 3), Parvovirus (4%, n = 2) and VZV (2%, n = 1) were detected respectively. Both enterovirus and HHV type 7 were detected in one patient. Genotyping was not performed on samples that were studied qualitatively by polymerase chain reaction. A patient with no viral agent in the CSF sample had meningococcal growth in the blood culture and ceftriaxone was administered to the patient for 7 days. The most common symptoms in cases with viral agents were 78% fever and 46% convulsion, respectively.

After the application evaluation, 11.2% (n = 21) intensive care follow-up was required. In the application period, 57.5% positivity was detected in C-reactive protein values of acute phase reactants. Comparison of values in CSF samples taken with lumbar puncture after application is shown in Table 2.

**Table 2:** Distribution of C-reactive protein and CSF laboratory findings and Evaluation of imaging results.

	Enterovirus (n = 12)	Adenovirus (n = 11)	HHV type6 (n = 11)	HHV type7 (n = 5)	EBV (n = 3)	HSV type 1 (n = 3)	CMV (n = 3)	Negative (n = 136)
C reactive protein positivity	83.3% (n = 10)	54.5% (n = 6)	45.5% (n = 5)	80% (n = 4)	66.6% (n = 2)	33.3% (n = 1)	33.3% (n = 1)	55.8% (n = 76)
Anormal BOS WBC count	83.3% (n = 10)	27.2% (n = 3)	18.1% (n = 2)	80% (n = 4)	66.6% (n = 2)	0% (n = 0)	66.6% (n = 2)	27.2% (n = 37)
Iıncreased CSF protein by age	33.3% (n = 4)	27.2% (n = 3)	36.3% (n = 4)	60% (n = 3)	66.6% (n = 2)	66.6% (n = 2)	0% (n = 0)	26.4% (n = 36)
**Imagining**								
CT	2	3	6	1	1	0	3	68
Normal	2	3	6	1	1	0	3	68
Anormal	0	0	0	0	0	0	0	0
MRI	8	7	6	4	2	3	2	66
Normal	7	6	6	2	0	2	2	61
Anormal	1	1	0	2	2	1	0	5
Transfontanel USG	2	0	0	0	0	0	0	5
Normal	2	0	0	0	0	0	0	5
Anormal	0	0	0	0	0	0	0	0
EEG	1	3	1	0	0	1	2	20
Normal	1	0	1	0	0	0	2	6
Anormal	0	3	0	0	0	1	0	14

*HHV* Human Herpes Virus, *CMV* Cytomegalovirus, *HSV* Herpes Simplex Virus, *EBV* Ebstein Barr Virus, *CT* Computed tomograpy, *MRI* Magnetic resonance imagining, *USG* Ultrasonograpy, *EEG*:Electroencephalograpy.

The diagnosis stage of the patients who met the possible, probable and confirmed definitions of meningoencephalitis was also evaluated retrospectively. The distribution of the used cranial computed tomography (CT), cranial magnetic resonance imagining (MRI), transfontanel ultrasonography (USG) techniques by patient groups are given in Table 2.

In the empirical treatment of the patients, antibiotherapy was preferred at 48.6%, antiviral treatment at rate of 4% and both of antibiotic and antiviral treatment at rate of 47.3%. After treatment with hospitalization, 94.6% of the patients were discharged with healing. Mortality and sequela rate resulted 0.53% (n = 1) and 3.7%, respectively.

## Discussion

A drastic change in the aetiology of childhood meningoencephalitis has occurred in Turkey since vaccination programmes have eradicated mumps, measles and rubella associated encephalitides. There is no study involving the national incidence rates of viral meningoencephalitis, except for Ceyhan et al.’s studies that have been following bacterial meningitis surveillance nationally for years^5^. We identifed a considerable number of associated agents, nine different viruses in 50 of 186 cases. A range of childhood encephalitis incidence rates have been reported in international studies, from 2.8 to 10.5 per 100,000 in England, Sweden and the USA, with highest rates inthe under 1 year age group (13.7 to 18.4 per 100,000)^6–8^.

In Europe and North America, enteroviruses are the most common causes of viral meningitis in children, accounting for 10 to 20% of cases in published series^8,9^. The present frequencies 24%. Enteroviruses have a clear seasonality, with 78 percent of cases in the United States occurring from June through October^10^. Similarly, in our study, we found that most of our cases clustered in the late spring and summer.

Meningoencephalitis of variable severity can rarely occur as a complication of the primary manifestation of HHV type 6 infection^11^. In tis study, HHV type 6 have 22% in frequency particularly in younger age groups. Also HHV type 7 was detected in 5/50 patients with meningoencephalitisat a later age than infection with human herpes virüs type 6 (73 months versus 23 months). The availability of multiplex polymerase chain reaction (PCR)-based tests has enhanced identification of causative agents for meningitis and encephalitis. Identification of HHV type 6 with multiplex PCR-based tests is not sufficient evidence of causation^12^. It is suggested confirmation that an illness is caused by HHV type 6 typically requires a compatible clinical syndrome, evidence of active HHV type 6 infection with viral replication. It is noteworthy that HHV type 6 and HHVtype 6 are high percent.

Meningitis and encephalitishave been reported occasionally in association with adenovirus infection^13^. Patients presented with fever, convulsions, diarrhea, and changing consciousness without upper and lower respiratory tract infections sypmtoms. The most striking feature of this study was that adenovirus was proportionally in the foreground.

VZV is one of the common causes of childhood encephalitis^14^. Since incorporation of varicella vaccine to well-child immunization schedule in 2013, varicella-related hospitalizations and deaths has declined in Turkey^15^. This supports the evidence that varicella vaccine reduces severe complications from varicella infection. Similarly, decreased incidence of varicella-related neurological disease was observed with childhood varicella immunization in Canada^16^. In support to these studies, our current study demonstrated a significant decline in the hospitalization rate of varicella encephalitis after start of routine vaccination in Turkey, for the first time at population level.

Herpes simplex virus encephalitis is a serious infectious disease,which can, despite appropriate treatment, lead to severeneurologic sequelae^17^. At onset, neurologic complaints such as fever, headaches, fatigue, and convulsions were dominating. Withregard to our data, the combination of the specific symptomsfever and convulsion should always raise suspicion forherpes simplex virus encephalitis. MRI is one major diagnostic tool in children with suspectedherpes simplex virus encephalitis. Diffusion-weighted imagingincluding apparent diffusion coefficient map showed arestricted diffusion pattern in 1/3 patients studied.

Diagnostic methods in encephalitis have been changed. PCR or antigen assays from CSF need to be done urgently because neuroimaging or EEG are seldom positive, and restrictive in diagnosis. Using our strict criteria, viral PCR diagnosis was reached in 26.8%.

The clinical diagnosis of encephalitis is difficult. Focal orprolonged convulsions should always arouse suspicion of a specific cause, especially of adenovirus and HHV type 6 infection. Ataxia, confusion, somnolence, headache and depressed consciouslevel, are highly suggestive of encephalitis. In theyoungest children the symptoms may be subtle. It is necessary to pay attention to the accompanying of gastroenteritis, rash.

In conclusion, we identified a cause in 50/186 (26.9%) suspected CNS infection cases using molecular diagnostic method. The three most common pathogens in children were enterovirus, adenovirus and HHV type 6. We also found that mortality rate was %0.53 (n = 1). Further prospective research is required, in the context of enhanced surveillance, to better define the aetiology ofchildhood encephalitis especially given the high proportion of ‘unknown’ encephalitis in our region. This highlights the importance of monitoring trends in encephalitis morbidity and mortality in Turkey with aview to improving pathogen diagnosis for encephalitis and rapidly identifying novel emerging encephalitis-causing pathogens that demand public health action especially in national immunisation programme.
